# Genetic Diversity and Phylogeography of the Important Medical Herb, Cultivated Huang-Lian Populations, and the Wild Relatives *Coptis* Species in China

**DOI:** 10.3389/fgene.2020.00708

**Published:** 2020-07-03

**Authors:** Xin Wang, Xiao-Quang Liu, Ya-Zhu Ko, Xiao-Lei Jin, Jia-Hui Sun, Zhen-Yu Zhao, Qing-Jun Yuan, Yu-Chung Chiang, Lu-Qi Huang

**Affiliations:** ^1^State Key Laboratory Breeding Base of Dao-di Herbs, National Resource Center for Chinese Materia Medica, China Academy of Chinese Medical Sciences, Beijing, China; ^2^Tianjin University of Traditional Chinese Medicine, Tianjin, China; ^3^Department of Biological Sciences, National Sun Yat-sen University, Kaohsiung City, Taiwan; ^4^Department of Biomedical Science and Environmental Biology, Kaohsiung Medical University, Kaohsiung City, Taiwan

**Keywords:** cultivated Huang-lian, wild *Coptis* species, plastid DNA, genetic diversity, phylogeography

## Abstract

Huang-lian (*Coptis* plants in China) are essential medicinal plants in China, *C. chinensis* var. *chinensis* and *C. deltoidea* have been domesticated and cultivated for 700 years. In this study, the genetic diversity patterns and biogeographical information of cultivated Huang-lian and their wild relatives *Coptis* species were assessed using three plastids DNA regions. A total of 186 individuals from twenty-seven populations representing two species of cultivated Huang-lian and four species of wild relatives were collected and analyzed. Twenty-four haplotypes of six species were identified when three plastid spacers were combined. Historical biogeography inference revealed multiple dispersal events in the groups of cultivated Huang-lian and *C. omeiensis*. This evidence can infer that large initial population size and interbreeding with co-existing wild relatives in expanding new planting areas might be the main reason for maintaining the high genetic diversity of cultivated Huang-lian. Nevertheless, the multimodal curve of mismatch analysis and positive or negative differed among species and populations by neutrality tests indicated some groups of cultivated Huang-lian experienced genetic bottlenecks. Phylogeny analysis (NJ, MP, BI) showed that cultivated Huang-lian and *C. omeiensis* were clustered into a monophyletic group while *C. chinensis* var. *brevisepala* was paraphyletic, having earlier divergence time from *C. chinensis* var. *chinensis* (7.6 Ma) than *C. omeiensis.* Parsimony network demonstrated that *C. deltoidea* had more shared haplotypes with *C. omeiensis* than *C. chinensis* var. *chinensis*, and other haplotypes of *C. deltoidea* and *C. omeiensis* had less mutation steps than that of *C. chinensis* var. *chinensis* and *C. omeiensis.* This evidence suggests that *C. omeiensis* has a closer relationship with cultivated Huang-lian and might be a potential wild relative to *C. deltoidea*. The results reported here provide the baseline data for preserving genetic resources of Huang-lian and also evaluating the genetic impacts of long-term cultivation on medicinal plants, which could be instructive to future cultivation projects of traditional Chinese medicinal plants.

## Introduction

Wild plant populations are subjected to domestication and selective cultivation around 12,000–10,000 years ago in the Fertile Crescent and elsewhere (Xu, 2010). Cultivated plant species experienced long periods of artificial selection and isolation transferring to new cultivars and types to suit human needs (Doebley et al., 2006). In some cases, taxonomists have divided these wild and domesticated plants into two distinct species, such as *Oryza sativa* and *O. rufipogon*, *Zea mays* and *Z. mexicana* (Huang et al., 2012; Niazi et al., 2015). However, the domestication of the wild plant might have reduced genetic diversity (Allaby et al., 2019). Founder effect and artificial selection for high yielding or other good agronomic traits, have resulted in a narrow genetic material in cultivation and have usually been examined in cereal species (Doebley et al., 2006) such as maize (Tenaillon et al., 2004), wheat (Haudry et al., 2007), and rice (Londo et al., 2006). Therefore, it is essential to understand the genetic diversity of wild plant populations, as wild plant resources provide infinite potentials for superior breeding varieties to improve the genetic diversity of cultivated plants.

Cultivation of medicinal plants has been documented for more than 2000 years, which is shorter than crop domestication (12,000 years ago) (Xiao, 1991; Olsen and Gross, 2008). Medicinal plants contain 12.5% of the 422000 plant species documented worldwide playing a central role not only as traditional medicines systems but also as trade-in herbal medicinal products (Schippmann et al., 2002). In China, there are at least 6000 medicinal plants (Xiao, 1991; Huang, 2011). Of these, more than 600 plant species are commonly used in Chinese medicine, and approximately 300 species are cultivated with commercial use (Xiao, 1991; Bodeker et al., 1997). The demand for traditional Chinese medicine is increasing rapidly and is being cultivated on a large scale (Li et al., 2015). However, large-scale cultivation faces challenges of plant pests and diseases, heavy metal, agrochemicals residues and biodiversity conservation (Li et al., 2015).

The strength of the bottleneck effect of domesticated species is determined by the bottleneck’s duration and adequate population size of the bottlenecked population (Olsen and Gross, 2008). Several previous studies have pointed out that nearly all of the crops have experienced bottleneck effects and loss of genetic diversity relative to their wild species during long-term domestication (Hyten et al., 2006; Yamasaki et al., 2007; Kovach and Mccouch, 2008). The effect of domestication bottlenecks also revealed different patterns in perennial and annual cultivated crops (Miller and Gross, 2011). Compared with their wild population, perennial cultivated fruit crops preserve a greater proportion of genetic variation than annual cultivated fruit crops since juvenile phase length, mating system, mode of reproduction, geographic origins of cultivated individuals, widespread hybridization, and crop-wild gene flow (Miller and Gross, 2011). For these recently cultivated medicinal plants (about several decades), such as *Scutellaria baicalensis* and *Gastrodia elata*, experimental evidence indicates that a slight genetic bottleneck since there is not sufficient time for artificial selection and extensive seed exchange (Yuan et al., 2010; Chen et al., 2014). Compared with the recently cultivated perennial medicinal plants, the information about whether there is a stronger genetic bottleneck for the long-term domesticated perennial medicinal plant is poor. Thus, it is necessary to investigate the genetic diversity patterns of long-term cultivated medicinal plants and to elucidate the relationship with wild relatives.

Huang-lian (*Rhizoma coptidis*), is the rhizome of *Coptis* plants. *Coptis* is a pharmaceutically herbaceous perennial genus of Ranunculaceae (Yu et al., 2006). In China, there are six species of *Coptis* [*C. chinensis* var. *chinensis*, *C*. *chinensis* var. *brevisepala* W. T. Wang and P. K. Hsiao, *C. omeiensis* (Chen) C. Y. Cheng, *C. deltoidea* C. Y. Cheng and P. K. Hsiao, *C. teeta* Wallich, *C. quinquesecta* W. T. Wang] and wild populations of these species are restricted distribution, mainly distributed in Southern and Southwest of China and Himalayas (Tamura, 1995; Yu, 1999; Fu and Orbélia, 2001; Xiang et al., 2016, 2018). In Taiwan, there is one *Coptis* species (*C. quinquefolia* Miquel) with small population grow on restricted habitat in northern Taiwan (Tamura, 1995; Fu and Orbélia, 2001; Xiang et al., 2018). Huang-lian is well-known for its berberine-rich and broad antibacterial activities. Pharmacopeia-recorded Huang-lian derived from three *Coptis* species: *C. chinensis* var. *chinensis*, *C. deltoidea* and *C. teeta* (China, 2015), which is called official Huang-lian. The other *Coptis* species are used as alternative Huang-lian in folk. *C. chinensis* var. *chinensis*, *C. omeiensis* and *C. teeta* are diploid (2*n* = 2*x* = 18), whereas *C. deltoidei* is auto triploid (2*n* = 3*x* = 27) (Pandit and Babu, 1993; Xiang et al., 2010; Huang et al., 2013). *C. chinensis* var. *chinensis* and *C. omeiensis* are propagated by seeds and no stolons (Song et al., 2010). *C. deltoidei* is propagated by stolons while is cannot form seeds (Li et al., 2010). *C. teeta* is propagated by seeds and stolons (Selvam and India, 2012). In China, the wild resources of these species are almost endangered (He et al., 2014; Zhong et al., 2018). *C. omeiensis* has not been artificially cultivated, and the source all originated from wild populations in China (Huang, 2012). *C. omeiensis* was listed as a national second-class endangered plant in China (Huang, 2012). *C. teeta* had been listed in the International Union for Conservation of Nature’s Red List (IUCN Red List) as Endangered (Saha et al., 2015). *C. teeta* has been cultivated and domesticated on a small scale (Mukherjee and Chakraborty, 2019). Compared with wild *C. teeta*, the cultivated *C. teeta* has been observed obvious morphological variations (Mukherjee and Chakraborty, 2019). *C. chinensis* var. *chinensis* and *C. deltoidea* have been artificially cultivated since the Yuan and Ming Dynasty (Xiong et al., 2011). Currently, *C. chinensis* var. *chinens* is cultivated in Chongqing, Hubei and Sichuan provinces (Lv et al., 2016). *C. deltoidea* is cultivated on both sides of Emeishan in Sichuan province (Xiong et al., 2011). *C. chinensis* var. *chinens* is a common and high-yielding material of Huang-lian. The information on the distribution, ploidy level, propagation and present cultivation status of six *Coptis* species are listed in Supplementary Table S1.

Huang-lian was first recorded in the earliest monograph on Chinese *material medical*, *Sheng Nong’s Herbal Classic* during the Eastern Han Dynasty (25–220 AD) (Yang, 1998; Wu, 2005). Huang-lian has been used by Chinese medicinal physicians for over 2000 years and has been domesticated and cultivated for 700 years in China (Tan et al., 2016). Therefore, it is an ideal model for exploring the effects of long-term domesticated and cultivated medicinal plants on genetic diversity patterns. Cultivated Huang-lian has abundant wild relative species resources. *C. teeta* is an endemic endangered plant of Eastern Himalaya. It is only reported from Indian Territory of Arunachal Pradesh, Burma, northwest Yunnan and Tibet provinces of China (Payum, 2017; Wang et al., 2019). The distribution of *C. teeta* does not overlap with *C. chinensis var. chinensis* and *C. deltoidea*. Wild *Coptis* of *C. omeiensis* is mainly distributed on Emeishan in Sichuan province, overlapping with the distribution of *C. deltoidea*. *C. chinensis* var. *brevisepala* is a wild plant distributed in the Southern Anhui mountainous areas, partially overlapping with the distribution of *C. chinensis* var. *chinensis* (Fu and Orbélia, 2001; Zhang and Zhang, 2005; Wang and Peng, 2008). *C. quinquesecta* is a critically endangered wild species native in southeastern Yunnan Province (Zhang et al., 2018), and has not found according to herbaria records.

Based on plastid and ITS DNA regions evidence, *C. quinquefolia* in Taiwan has a relatively distant relationship with the other six *Coptis* species in China (Xiang et al., 2016). Furthermore, *C. chinensis* var. *chinensis* and *C. chinensis* var. *brevisepala* are not clustered in a group. Therefore, Xiang et al. suggest that *C. chinensis* var. *brevisepala* should be regarded as a species rank (Xiang et al., 2016). According to metabolic and molecular genetic information, *C. omeiensis* and *C. deltoidea* had the closest genetic relationship among *Coptis* plants (He et al., 2014; Xiang et al., 2016; Chen et al., 2017; Zhong et al., 2018). There are only a few molecular phylogeny studies of genus *Coptis* have been reported so far (He et al., 2014; Xiang et al., 2016; Chen et al., 2017; Zhong et al., 2018). Only some researches have been done to gain information about the genetic diversity of *Coptis* species using molecular methods such as Inter-simple sequence repeats (ISSR) (Chen et al., 2006; Shi et al., 2008). However, less attention was paid to the comprehensive genetic evaluation of *Coptis* species with medicinal effects. There is urgent to explore the genetic information between long-term domesticated Huang-lian and their wild populations or wild relative species.

Nowadays, the assessment of genetic diversity is mainly based on DNA-based methods, which can be classified into fragment analysis-based methods, hybridization array-based methods, and sequencing-based methods (Loera-Sánchez et al., 2019). The use of such methods to assess the genetic diversity will require careful evaluation and balance of the biological features of the plant species, and the strengths and limitations of the method used, in order to optimally exploit suitable techniques for study genetic diversity. Among them, using amplicon sequencing to assess the genetic diversity is a low-cost and complexity reduction alternative method since only standard PCR reagents are required. It has been applied to assess the genetic diversity information and a monitoring tool for mixture breeding in various plant species (Loera-Sánchez et al., 2019). Therefore, this study employs DNA sequencing technologies to assess the phylogenetic relationships and genetic diversity of Huang-lian and wild relatives, which can help to establish and monitoring genetic resource conservation and can also support the species registration.

Phylogeography integrates genealogical and geographical information to understand the demographic and historical processes that shaped the evolution of populations or closely related species (Avise, 2000; Kuchta and Meyer, 2001). Phylogeographical approaches are well suited for understanding the domestication processes and history because domesticated species usually include many different degrees of divergence in the evolutionary lineages resulting from the several population recent differentiation processes (Aguirre-Dugua and González-Rodríguez, 2016). Here, we applied three plastids and one nuclear (ITS) DNA regions to investigate the genetic variability and phylogenetic relationships of six *Coptis* species and analyzed the biogeographical information to clarify the evolutionary process. The objectives of this study are (1) to evaluate the genetic diversity pattern of cultivated Huang-lian; (2) to elucidate the phylogenetic and phylogeographical relationships among wild relatives *Coptis* species and cultivated Huang-lian

## Materials and Methods

### Plant Material Sampling

A total of 248 individuals from twenty-seven populations were collected and analyzed in this study (Table 1), including *C. chinensis* var. *chinensis* (Ccc: 38 individuals from 5 cultivated populations), *C. deltoidea* (Cd: 46 individuals from 5 cultivated populations), *C. chinensis* var. *brevisepala* (Ccb: 51 individuals from 4 wild populations), *C. teeta* (Ct: 38 individuals from 5 wild populations), *C. omeiensis* (Co: 65 individuals from 7 wild populations) and *C. quinquefolia* (Cq: 10 individuals from 1 wild population). This study contained most of the *Coptis* species in Taiwan and China but did not include *C. quinquesecta*, because the species is a critically endangered medicinal plant and was not found in the field. Fresh leaves of *Coptis* species were collected and dried with silica gel for DNA extraction. Voucher specimens were deposited in herbaria of Institute of Chinese *Materia Medica* (CMMI), China Academy of Chinese Medical Sciences. The precise geographic location of each sampled population was determined using a Garmin GPS unit (Table 1). The abbreviation naming rules are follows: species abbreviation + (population abbreviation) + W (wild) or C (cultivated).

**TABLE 1:** Details of sample locations, sample sizes and the number of individuals and clones sequenced of cpDNA and ITS.

Taxon	Locality	Population code	Latitude / longitude	Sample size	No. individuals or clones			
					cpDNA*	ITS*	ITS†	ITS‡
*C. chinensis* var. *brevisepala*	Huanjiang, Guangxi	Ccb(HJ)W	24°49′48″ N/108°15′36″ E	11	8	9	2	25
	Shexian, Anhui	Ccb(SX)W	30°05′11″ N/118°18′58″ E	21	21	21	0	0
	Jinggangshan, Jiangxi	Ccb(JGS)W	26°31′02” N/114°09′59” E	13	13	7	0	0
	Tonggu, Jiangxi	Ccb(TG)W	28°29′60” N/114°24′28” E	6	5	6	0	0
*C. chinensis* var. *chinensis*	Emeishan, Sichuan	Ccc(EMS1)C	29°34′10” N/103°16′22” E	9	4	9	0	0
	Emeishan, Sichuan	Ccc(EMS2)C	29°34′10” N/103°21′19” E	7	5	0	7	47
	Xuanen, Hubei	Ccc(XE)C	30°11′27” N/109°46′31 ”E	8	7	0	8	30
	Jinfoshan, Chongqing	Ccc(JFS)C	29°09′28” N/107°05′57 ”E	8	7	8	0	0
	Dazu, Chongqing	Ccc(DZ)C	29°27′00” N/105°35′24” E	6	6	0	0	0
*C. deltoidea*	Emeishan, Sichuan	Cd(EMS1)C	29°34′42” N/103°21′41” E	9	5	9	0	0
	Emeishan, Sichuan	Cd(EMS2)C	29°34′12” N/103°21′57” E	8	7	0	8	59
	Hongya, Sichuan	Cd(HY)C	29°29′59” N/103°14′56” E	8	5	3	8	39
	Emeishan, Sichuan	Cd(EMS3)C	29°34′20” N/103°24′07” E	13	5	11	2	7
	Emeishan, Sichuan	Cd(EMS4)C	29°34′19” N/103°24′07” E	8	8	0	5	20
*C. omeiensis*	Emeishan, Sichuan	Co(EMS1)W	29°34′02” N/103°17′09” E	6	5	6	0	0
	Emeishan, Sichuan	Co(EMS2)W	29°34′42” N/103°21′41” E	13	13	8	4	26
	Emeishan, Sichuan	Co(EMS3)W	29°34′42” N/103°21′42” E	7	6	7	0	0
	Emeishan, Sichuan	Co(EMS4)W	29°34′39” N/103°17′50” E	11	8	11	0	0
	Emeishan, Sichuan	Co(EMS5)W	29°34′49” N/103°21′51” E	13	3	13	0	0
	Emeishan, Sichuan	Co(EMS6)W	29°35′23” N/103°21′19” E	5	3	5	0	0
	Emeishan, Sichuan	Co(EMS7)W	29°34′19” N/103°24′07” E	10	7	10	0	0
*C. teeta*	Gaoligongshan, Yunnan	Ct(GLGS1)W	27°43′24” N/ 98°39′41” E	7	7	5	0	0
	Gaoligongshan, Yunnan	Ct(GLGS2)W	27°43′25” N/ 98°37′12” E	5	5	5	0	0
	Fugong, Yunnan	Ct(FG1)W	27°21′15” N/ 98°28′52” E	11	6	9	2	25
	Fugong, Yunnan	Ct(FG2)W	26°19′25” N/ 98°34′56” E	5	5	5	0	0
	Fugong, Yunnan	Ct(FG3)W	26°33′30” N/ 98°55′42” E	10	5	10	0	0
*C. quinquefolia*	Yilan, Taiwan	Cq(YL)W	24°27′00” N/121°27′00” E	10	7	10	0	0
Total				248	186	187	46	278

*No. individuals directly sequenced; †No. individuals cloned; ‡No. clones sequenced; the last letter of population code: W: wild, C: cultivated. The bold and red letter indicates that the sample is from cultivated.

### DNA Extraction, PCR Amplification, and Sequencing

Genomic DNA was extracted using a modified cetyltrimethylammonium bromide (CTAB) protocol (Doyle and Doyle, 1987). Four DNA-barcoding regions recommended by China Plant BOL Group, i.e., chloroplast *trn*H-*psb*A, *rbc*L, *mat*K, and nuclear ribosomal (nr) ITS were amplified and sequenced (Li et al., 2011). The primers suggested for *rbc*L (1F-724R) and ma*t*K (3F-1R) by the CBOL Plant Working Group (2009), *trn*H-*psb*A by Sang et al. (1997), and ITS (ITS5-ITS4) by White et al. (Innis et al., 1990) were used in this study. DNA amplification was performed in a TC-512 thermocycler (Techne, United Kingdom), programmed for a primary denaturing step at 94°C for 240 s, followed by 35 cycles of 45 s at 94°C, annealing at a specific temperature for 30 s and extension 90 s at 72°C, with a final extension of 10 min at 72°C. Reactions were carried out in a volume of 20 μL containing 2.0 mmol/L MgCl_2_, 0.5 μmol/L dNTP, 10 × buffer, 2.5 μmol/L primer, 1.0 U Ex*-Taq* DNA polymerase and 20 ng DNA template.

Sequencing reactions were conducted with the forward or reverse primers of the amplification reaction using the DYEnamic ET Terminator Kit (Amersham Pharmacia Biotech) following the manufacturer’s protocol. Sequencing was performed on an ABI 377XL automated DNA sequencer after the products of the direct sequencing reactions were purified through precipitation with 95% ethanol and 3 M sodium acetate (pH 5.2).

For those PCR products of ITS failed to directly sequence, some of them were cloned into the pGEM-T easy-cloning vector (TaKaRa) to separate multicopy or allele within the individual. Two to eight clones each PCR product were selected and reamplified. The direct cycle sequencing reactions were performed using a Thermo Sequenase cycle sequencing kit (rpn2438 from Amersham Pharmacia Biotech) with IRD700- and IRD800-labeled primers (MWG Biotech, Ebersberg, Germany). Sequencing was performed on a LICOR L4200S-2 automated sequencer.

### Data Analysis

All the sequences were edited and assembled by using C**ONTIGEXPRESS** (**VECTOR NTI ADVANCE** version 10.3.1, Invitrogen) and B**IOEDIT** 7.0.9 to assisted with manual sequence correction. Refer to the methods of Caicedo and Schaal, nucleotide insertion and deletion (indel) were coded as substitutions (Caicedo and Schaal, 2004). Sequence alignments for each region were carried out using C**LUSTAL_**X version 2.0 (Larkin et al., 2007) and then checked by eye in B**IOEDIT**. Accessions from each species were incorporated into one or more private sequences to checking the shared alleles among species by using DAMBE (Xia and Xie, 2001). Before conducting all analyses, the incongruence length difference (ILD) test (Farris et al., 1994) was conducted to evaluate congruency between data from the three cpDNA regions implemented in PAUP^∗^ 4.0b10 (Swofford, 2002).

Genetic diversity information was evaluated based on the number of segregating sites (*S*), the number of haplotypes (*h*), haplotype diversity (*Hd*) and nucleotide diversity (π) (Nei, 1987) using D**NA**SP 5.10.1 (Librado and Rozas, 2009). Tests of neutrality and determination of the associated significance were carried out based on Tajima’s *D* statistic (Tajima, 1989) and Fu and Li’s *D*^∗^ statistic (Fu and Li, 1993) using D**NA**SP 5.10.1 (Librado and Rozas, 2009). Pairwise *F*_ST_-values among populations and species were also estimated hierarchically in program D**NA**SP 5.10.1 (Librado and Rozas, 2009). The genetic structure was evaluated to estimate the distinction among species in *Coptis* by AMOVA analysis with ARLEQUIN version 3.1 (Excoffier et al., 2005), partitioning the genetic diversity into three levels: among species, among-population within species and within-population. Significance tests were conducted using 10,000 permutations. A statistical parsimony haplotype network estimated the genetic relationships among haplotypes by using **TCS** 1.21 (Clement and Crandall, 2000).

### Phylogenetic Analyses

Phylogenetic relationships of haplotypes were inferred using neighbor-joining (NJ), Maximum parsimony (MP) and Bayesian Inference (BI) methods at the same time. NJ phylogenetic tree was constructed by using MEGA X software under the Tamura 3-parameter model (Kumar et al., 2018). MP phylogenetic tree analyses were carried out in PHYLIP Version 3.695 (Felsenstein, 1993), calculated by the modules SEQBOOT, DNAPARS, and CONSENSE. Phylogenetic trees were generated by maximum parsimony (MP) methods in the DNAPARS program, SEQBOOT program (1000 replicates) for generating bootstrap values and CONSENSE program to find a consensus tree. For Bayesian analysis, we chose Bayesian methods implemented in M**R**B**AYES** 3.1.2 (Huelsenbeck and Ronquist, 2001; Ronquist and Huelsenbeck, 2003) with the best-fitting GTR+I+G model selected by MODELTEST 3.7 (Posada, 1998) using the Akaike information criterion (Akaike, 1974). Four chains, each with a different starting seed, were run for two million generations; each analysis was repeated twice. Trees were subsequently sampled every 100 generations. Stationarity was reached after approximately 400,000 generations, therefore, the first 4000 trees were discarded as burn-in. The general criteria for determining stationarity has been reached was when the value of the likelihood of the sample points reached a stable equilibrium (Huelsenbeck and Ronquist, 2001). The value was the standard deviation of the split frequencies was less than 0.01, with the potential scale reduction factors for each parameter was close to 1. Bayesian posterior probabilities were estimated as the proportion of trees containing each node relative to the total tree number sampled during runs.

Divergence time estimation among and within species was carried on B**EAST** 1.7.2 (Drummond and Rambaut, 2007). As a taxon-specific substitution rate had not been previously calibrated for non-coding cpDNA regions of *Coptis* species, we assumed a substitution rate of 1.52 × 10^–9^ substitutions per neutral site per year (s/s/y) and set 1.0 × 10^–9^ and 3.0 × 10^–9^ as the lower and the upper limit for nucleotide substitution rate, respectively (Wolfe et al., 1987). Three independent Markov chain Monte Carlo (MCMC) pre-runs of ten million generations were performed. Genealogies were sampled every 1000 generations with the first 10% discarded as burn-in. Statistics of the output values were summarized using Tracer 1.5 (Rambaut and Drummond, 2009), and both log and tree files were combined using LOGCOMBINER 1.6.1 (Drummond and Rambaut, 2007). We applied TREEANNOTATOR 1.6.1 (Drummond and Rambaut, 2007) and FIGTREE 1.4 (Rambaut, 2012) to summarize and display the sampled trees, respectively.

Bayesian skyline plot (BSP) was created to infer the historical demography of all species using by running BSP analysis using the software B**EAST** 1.7.2. A GTR+I+G model was selected with an assuming nucleotide substitution rate of 1.52 × 10^–9^ s/s/y. Three independent Markov chains were run for 2.0 × 10^7^ generations and were sampled every 1000 generations discarding the first 10% as burn-in. Mismatch analysis was performed to evaluate the range expansions of the *Coptis* species under the sudden expansion model and the spatial expansion model using Arlequin ver 3.5 (Excoffier and Lischer, 2010). The sum of squared deviations (SSD) was used as a statistical method to test the validity of the expansion model. *P*-values were then calculated as the proportion of simulations generating an SSD more substantial than the observed SSD. The raggedness index (HRag) and its significance for observed distributions were used to compute the smoothness of the mismatch distributions (Harpending, 1994). Low raggedness implied recently non-stationary, expanding populations.

### Phylogeographical Inference Using RASP

To reveal the biogeographical history, the origin and mechanism (vicariance or dispersal) of the geographical distribution of *Coptis*, statistical dispersal-vicariance analysis (S-DIVA) and Bayesian binary MCMC (BBM) analysis performed using RASP (Reconstruct Ancestral State in Phylogenies) (Yu et al., 2015). The geographical distribution of the species was categorized into seven areas: (A) Yunnan province (5 populations of *C. teeta*), China; (B) Sichuan province (all the populations of *C. deltoidea* and *C. omeiensis*, and two populations of *C. chinensis* var. *chinensis*), China; (C) Taiwan Island (unique population of *C. quinquefolia*), China; (D) Anhui province (only Ccb (SX)W), China; (E) Guangxi province (only Ccb(HJ)W), China; (F) Chongqing municipality and Hubei province (three populations of *C. chinensis* var. *chinensis*), China; and (G) Jiangxi province (Ccb(JGS)W and Ccb(TG)W), China. The tree topologies generated by M**R**B**AYES** and B**EAST** were congruent in the major clades; therefore, 20,000 trees from the B**EAST** MCMC outputs were used, and the BEAST annotated tree was set as the final condensed tree. The number of maximum areas at each node allowed in ancestral distributions was kept as three. The other parameters were automatically optimized.

## Results

### Sequence Characteristics of Chloroplast DNA and ITS

A total of 186 individuals representing twenty-seven populations of six species or variation of *Coptis* were successfully amplified and sequenced using chloroplast *trn*H-*psb*A, *rbc*L and *mat*K. The newly generated sequences in this study were submitted to GenBank (Supplementary Table S2). Sequence lengths after alignment were 373, 669 and 727 bp for *trn*H-*psb*A, *rbc*L and *mat*K, respectively. The *mat*K region was the most variable (35 polymorphisms detected in 727 aligned positions; 4.81%), followed by *trn*H-*psb*A (17/373, 4.56%) and *rbc*L (9/669, 1.35%). The *mat*K and *trn*H-*psb*A region had 55 indels of 1−5 bp and 10 indels of 1−70 bp, respectively. The *rbc*L matrix had no indels. The results of the incongruence length difference (ILD) test suggested a high degree of homogeneity among the three cpDNA regions (*P* > 0.5), which therefore were combined and formed twenty-four haplotypes (Hap1-24) based on 51 segregating sites (Tables 1, 2 and Supplementary Table S2).

**TABLE 2:** Estimate of genetic variability of cpDNA.

Chloroplast DNA		*S*	*h*	*Hd*	π(×10^–3^)	Fu and Li′s D*	Tajima′s *D*
*C. chinensis* var. *brevisepala*		23	9	0.74	5.08	1.40	1.43
	Ccb(HJ)W	8	2	0.25	1.36	−1.86*	−1.70
	Ccb(SX)W	0	1	0.00	0.00	—	—
	Ccb(JGS)W	6	5	0.54	1.34	0.06	0.08
	Ccb(TG)W	0	1	0.00	0.00	—	—
*C. chinensis* var. *chinensis*		7	5	0.70	1.44	1.28	0.58
	Ccc(EMS1)C	2	3	0.83	0.68	−0.71	−0.71
	Ccc(EMS2)C	0	1	0.00	0.00	—	—
	Ccc(XE)C	2	3	0.67	0.58	−0.06	0.21
	Ccc(JFS)C	1	2	0.57	0.39	1.32	1.34
	Ccc(DZ)C	1	2	0.60	0.41	1.05	1.45
*C. deltoidea*		9	7	0.62	1.45	0.23	−0.19
	Cd(EMS1)C	2	3	0.80	0.68	0.24	0.24
	Cd(EMS2)C	0	1	0.00	0.00	—	—
	Cd(HY)C	1	2	0.40	0.27	−0.82	−0.82
	Cd(EMS3)C	0	1	0.00	0.00	—	—
	Cd(EMS4)C	1	2	0.25	0.17	−1.13	−1.05
*C. omeiensis*		5	3	0.53	1.42	1.11	2.05*
	Co(EMS1)W	0	1	0.00	0.00	—	—
	Co(EMS2)W	0	1	0.00	0.00	—	—
	Co(EMS3)W	0	1	0.00	0.00	—	—
	Co(EMS4)W	0	1	0.00	0.00	—	—
	Co(EMS5)W	0	1	0.00	0.00	—	—
	Co(EMS6)W	0	1	0.00	0.00	—	—
	Co(EMS7)W	5	2	0.57	1.94	1.42	1.98*
*C. teeta*		6	5	0.74	0.86	−1.07	−0.51
	Ct(FG3)W	0	1	0.00	0.00	—	—
	Ct(GLGS1)W	0	1	0.00	0.00	—	—
	Ct(GLGS2)W	1	2	0.60	0.41	1.22	1.22
	Ct(FG1)W	0	1	0.00	0.00	—	—
	Ct(FG2)W	3	2	0.40	0.81	−1.05	−1.05
*C. quinquefolia*							
	Cq(YL)W	0	1	0.00	0.00	—	—
Overall		51	24	0.91	5.71	1.32	−0.233

**S*: Number of segregating sites, *h*: Number of haplotypes, *Hd*: haplotype diversity, π: Nucleotide diversity. The bold and red letter indicates that the sample is from cultivated.*

ITS was difficult to sequence directly by PCR in some populations of *Coptis* species, especially in *C. chinensis* var. *chinensis* and *C. deltoidea*. Sequencing by clone was performed in most populations of the two species and a few populations of the remaining species. The sequences by PCR and by clone presented 174 haplotypes (H1-174) and were submitted to GenBank (Table 1 and Supplementary Table S2), which showed significant intragenomic variability (average of 3.78 polymorphisms per individual). The results suggest that ITS may be incomplete concerted evolution in *Coptis* and was not suitable for phylogenetic analyses and phylogeographical inference. Therefore, only chloroplast DNA sequences were used in the following analyses.

### Genetic Diversity and Differentiation of *Coptis* Species

**FIGURE 1:**
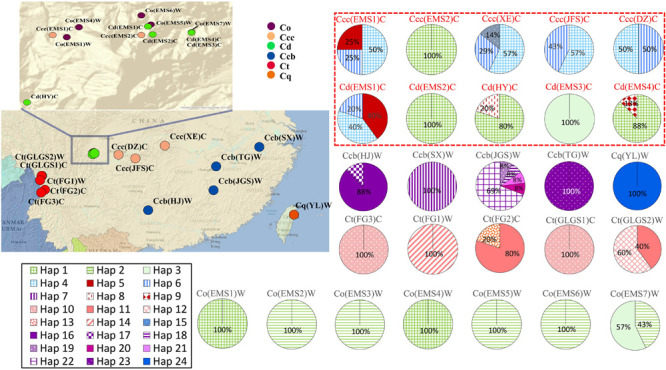
Geographic distribution and haplotype network of cpDNA haplotypes in populations of *C. chinensis* var. *brevisepala* (Ccb), *C. chinensis* var. *chinensis* (Ccc), *C. deltoidea* (Cd), *C. omeiensis* (Co), *C. teeta* (Ct), and *C. quinquefolia* (Cq). Pie charts show haplotype proportions for site locations. See Table 1 for abbreviation codes. The red letter indicates that the sample is from cultivated.

Genetic variability of cpDNA was estimated by the number of haplotypes, haplotype diversity and nucleotide diversity in *Coptis* species (Table 2). Haplotype Hap1, with the highest haplotype frequency (19.4%), was shared between cultivated Huang-lian, *C. chinensis* var. *chinensis* and *C. deltoidea*, and wild *C. omeiensis* (Figure 1 and Supplementary Table S2). *C. deltoidea* shared four haplotypes (Hap1, Hap4-6) with *C. chinensis* var. *chinensis* and two haplotypes (Hap1, Hap3) with *C. omeiensis*, respectively. Only two or one private haplotype were detected in the three species (*C. deltoidea*: Hap8-9, *C. chinensis* var. *chinensis*: Hap15, and *C. omeiensis*: Hap2). Among the remaining three species, no haplotypes were shared, nine (Hap7, 16-23), five (Hap10-14) and one (Hap24) private haplotypes were fixed in *C. chinensis* var. *brevisepala*, *C. teeta* and *C. quinquefolia*, respectively. The proportion of the population with unique haplotype in cultivated Huang-lian (*C. chinensis* var. *chinensis*: 1/5; *C. deltoidea*: 2/7) was lower than that in wild *Coptis* species (*C. omeiensis*: 1/4; *C. chinensis* var. *brevisepala*: 9/9; *C. teeta*: 5/5; *C. quinquefolia*: 1/1) (Figure 1).

**FIGURE 2:**
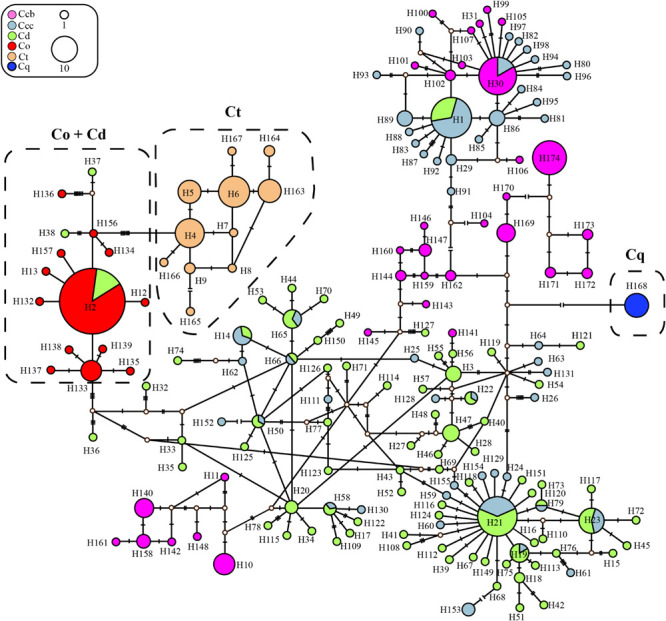
Parsimony network for all ITS haplotypes of *C. chinensis* var. *brevisepala* (Ccb), *C. chinensis* var. *chinensis* (Ccc), *C. deltoidea* (Cd), *C. omeiensis* (Co), *C. teeta* (Ct), and *C. quinquefolia* (Cq) species based on cpDNA data. The relative size of each circle corresponds to proportional to haplotype frequencies. The small hollow dots indicate hypothetical missing haplotypes. The network shown in different colors correspond to each species.

In the haplotype data of ITS, haplotype H2 with the highest haplotype frequency (16.0%), was shared between cultivated Huang-lian of *C. deltoidei* and wild *C. omeiensis* (Supplementary Tables S2, S3 and Figure 2). *C. deltoidea* shared eleven haplotypes (H1, H14, H19, H21-23, H50, H58, H65-66, H79) with *C. chinensis* var. *chinensis* and one haplotype (H2) with *C. omeiensis*, respectively. *C. chinensis* var. *chinensis* shared one haplotype (H30) with *C. chinensis* var. *brevisepala* (Figure 2 and Supplementary Tables S2, S3). *C. teeta* did not share any genotypes with other species. *C. deltoidei* had the largest number of private genotypes (Figure 2 and Supplementary Tables S2, S3).

Total haplotype diversity (*Hd*) and nucleotide diversity (π) in all species of *Coptis* were 0.91 and 0.00571, respectively (Table 2). Haplotype diversity and nucleotide diversity of cultivated Huang-lian (Ccc: 0.70, 0.00144; Cd: 0.62, 0.00145) was higher than that of *C. omeiensis* (0.53; 0.00142) and lower than that of *C. chinensis* var. *brevisepala* (0.74; 0.00508). However, the haplotype diversity (0.74) and nucleotide diversity (0.00086) of *C. teeta* were inconsistent. The former was higher while the latter was lower than those of cultivated Huang-lian. In summary, the genetic diversity of cultivated Huang-lian has not reduced compared with three wild *Coptis* species. However, the genetic diversity within populations of cultivated Huang-lian significantly raised. The top two *Hd* were observed in two cultivated populations Ccc(EMS1)C (0.83) and Cd(EMS1)C (0.80) in order. The *Hd* of *C. chinensis* var. *chinensis* (>0.57 except Ccc(EMS2)C) was higher than that of its wild variety, *C. chinensis* var. *brevisepala* (<0.54). The *Hd* of *C. deltoidea* (>0.25 except Cd(EMS2)C and Cd(EMS3)C) was higher than that of sympatric wild *Coptis* species, *C. omeiensis* ( = 0 except Co(EMS7)W). These results indicated human activities have increased a potential gene flows among cultivated populations during domestication.

Genetic differentiation between *Coptis* species was estimated based on *F*_ST_ (Table 3). The genetic differentiation between *C. deltoidea* and *C. omeiensis* (*F*_ST_ = 0.32) was the least in those between *C. deltoidea* and other species of *Coptis*. The genetic differentiation between *C. chinensis* var. *chinensis* and *C. chinensis* var. *brevisepala* (*F*_ST_ = 0.41) was the least in those between *C. chinensis* var. *chinensis* and other species of *Coptis*, but was higher than that between *C. deltoidea* and *C. omeiensis* (*F*_ST_ = 0.32). *C. quinquefolia* (*F*_ST_>0.69) and *C. teeta* (*F*_ST_>0.62) have significant divergence from other species of *Coptis* (Table 3). Among intraspecies populations, cultivated Huang-lian showed lower genetic differentiation and a certain degree of homogenization (Table 4). Only one population (Ccc (E2)) of *C. chinensis* var. *chinensis* (*F*_ST_>0.92) and two populations (Cd (E1) and Cd (E3)) of *C. deltoidea* (*F*_ST_>0.83) were significantly divergent from other intraspecies populations, while the remaining populations of the two species had no differentiation each other (*F*_ST_ = 0). Wild *Coptis* species with high haplotype diversity presented relatively high divergence among intraspecies populations, *F*_ST_-values ranging from 0.65 to 1.00 for the populations of *C. chinensis* var. *brevisepala*. *F*_ST_-values ranging from 0.25 to 1.00 for the populations of *C. teeta*, except for the differentiation between Ct (F3) and Ct (G1) populations (*F*_ST_ = 0) (Table 4). *F*_ST_-values ranging from 0.38 to 1.00 for the populations of *C. omeiensis*, except for the differentiation between Co (E1) and Co (E2), Co (E1) and Co (E4), Co (E2) and Co (E4), Co (E3) and Co (E5), Co (E3) and Co (E6), Co (E5) and Co (E6) populations (*F*_ST_ = 0) (Table 4).

**TABLE 3:** List of pairwise *F*_ST_-values among species deduced from cpDNA sequences.

	*C. chinensis var. brevisepala*	*C. chinensis var. chinensis*	*C. deltoidea*	*C. omeiensis*	*C. quinquefolia*	*C. teeta*
*C. chinensis* var. *brevisepala*						
*C. chinensis* var. *chinensis*	0.41					
*C. deltoidea*	0.56	0.50				
*C. omeiensis*	0.52	0.48	0.32			
*C. quinquefolia*	0.69	0.77	0.80	0.79		
*C. teeta*	0.62	0.79	0.85	0.84	0.83	

*The bold and red letter indicates that the sample is from cultivated.*

**TABLE 4:** List of pairwise *F*_ST_-values among populations deduced from cpDNA sequences.

	Ccb (HJ)	Ccb (JG)	Ccb (SX)	Ccb (TG)	Ccc (DZ)	Ccc (E1)	Ccc (E2)	Ccc (JFS)	Ccc (XE)	Cd (E1)	Cd (E2)	Cd (E3)	Cd (E4)	Cd (HY)	Co (E1)	Co (E2)	Co (E3)	Co (E4)	Co (E5)	Co (E6)	Co (E7)	Cq (YL)	Ct (F1)	Ct (F2)	Ct (F3)	Ct (G1)	Ct (G2)
Ccb (HJ)																											
Ccb (JG)	0.82																										
Ccb (SX)	0.94	0.91																									
Ccb (TG)	0.88	0.65	1.00																								
Ccc (DZ)	0.81	0.76	0.97	0.88																							
Ccc (E1)	0.79	0.75	0.95	0.83	0.00																						
Ccc (E2)	0.87	0.90	1.00	1.00	0.95	0.92																					
Ccc (JFS)	0.81	0.77	0.97	0.89	0.00	0.00	0.95																				
Ccc (XE)	0.79	0.75	0.96	0.84	0.00	0.00	0.93	0.00																			
Cd (E1)	0.80	0.75	0.96	0.84	0.00	0.00	0.92	0.08	0.00																		
Cd (E2)	0.87	0.90	1.00	1.00	0.95	0.92	0.00	0.95	0.93	0.92																	
Cd (E3)	0.89	0.91	1.00	1.00	0.95	0.91	1.00	0.95	0.92	0.91	1.00																
Cd (E4)	0.86	0.89	0.99	0.98	0.92	0.90	0.00	0.93	0.91	0.90	0.00	0.89															
Cd (HY)	0.85	0.88	0.99	0.97	0.91	0.89	0.00	0.92	0.89	0.89	0.00	0.83	0.00														
Co (E1)	0.87	0.90	1.00	1.00	0.95	0.92	0.00	0.95	0.93	0.92	0.00	1.00	0.00	0.00													
Co (E2)	0.87	0.90	1.00	1.00	0.95	0.92	0.00	0.95	0.93	0.92	0.00	1.00	0.00	0.00	0.00												
Co (E3)	0.83	0.86	1.00	1.00	0.91	0.88	1.00	0.92	0.88	0.88	1.00	1.00	0.97	0.95	1.00	1.00											
Co (E4)	0.87	0.90	1.00	1.00	0.95	0.92	0.00	0.95	0.93	0.92	0.00	1.00	0.00	0.00	0.00	0.00	1.00										
Co (E5)	0.83	0.86	1.00	1.00	0.91	0.88	1.00	0.92	0.88	0.88	1.00	1.00	0.97	0.95	1.00	1.00	0.00	1.00									
Co (E6)	0.83	0.86	1.00	1.00	0.91	0.88	1.00	0.92	0.88	0.88	1.00	1.00	0.97	0.95	1.00	1.00	0.00	1.00	0.00								
Co (E7)	0.67	0.74	0.90	0.79	0.63	0.60	0.38	0.63	0.61	0.61	0.38	0.33	0.33	0.34	0.38	0.38	0.50	0.38	0.50	0.50							
Cq (YL)	0.96	0.95	1.00	1.00	0.98	0.97	1.00	0.98	0.97	0.97	1.00	1.00	0.99	0.99	1.00	1.00	1.00	1.00	1.00	1.00	0.93						
Ct (F1)	0.92	0.89	1.00	1.00	0.95	0.93	1.00	0.96	0.94	0.93	1.00	1.00	0.99	0.98	1.00	1.00	1.00	1.00	1.00	1.00	0.87	1.00					
Ct (F2)	0.87	0.82	0.96	0.90	0.86	0.84	0.95	0.87	0.85	0.85	0.95	0.95	0.93	0.93	0.95	0.95	0.93	0.95	0.93	0.93	0.81	0.97	0.63				
Ct (F3)	0.92	0.90	1.00	1.00	0.96	0.94	1.00	0.96	0.94	0.94	1.00	1.00	0.99	0.98	1.00	1.00	1.00	1.00	1.00	1.00	0.88	1.00	1.00	0.63			
Ct (G1)	0.92	0.90	1.00	1.00	0.96	0.94	1.00	0.96	0.94	0.94	1.00	1.00	0.99	0.98	1.00	1.00	1.00	1.00	1.00	1.00	0.88	1.00	1.00	0.63	0.00		
Ct (G2)	0.90	0.86	0.98	0.95	0.92	0.89	0.97	0.92	0.90	0.90	0.97	0.98	0.96	0.96	0.97	0.97	0.97	0.97	0.97	0.97	0.85	0.99	0.81	0.25	0.81	0.81	

*[Species code] Ccb: *C. chinensis* var. *brevisepala*; Ccc: *C. chinensis* var. *chinensis*; Cd: *C. deltoidea*; Co: *C. omeiensis*; and Ct: *C. teeta.* [Population code] JG: JGS; TG: TGS; E1: EMS1; E2: EMS2; E3: EMS3; E4: EMS4; E5: EMS5; E6: EMS6; E7: EMS7; F1: FG1; F2: FG2; F3: FG3; G1: GLGS1; G2: GLGS2. See Table 1 for abbreviation codes.*

Hierarchical AMOVA based on cpDNA data revealed that 5.49% of the total variance was distributed within populations (Φ*_ST_* = 0.95, *P* < 0.05), and a considerable amount of variation originated from differentiations among populations within species (38.15%, Φ*_SC_* = 0.87, *P* < 0.05) and over a half of all variance distinguished different species from each other (56.36%, Φ*_CT_* = 0.56, *P* < 0.05) (Table 5). For each species, *C. chinensis* var. *chinensis* had the lowest genetic variation among populations (Φ*_ST_* = 0.75) and *C. chinensis* var. *brevisepala* had the highest genetic variation among populations (Φ*_ST_* = 0.92).

**TABLE 5:** Results of different hierarchical types analysis of molecular variance (AMOVA) of chloroplast DNA sequence data.

	Sum of squares	*d.f.*	Variance components	Percentage of variation	Fixation Indices
**All species**					
Among species	478.47	5	2.71	56.36	Φ*_CT_* = 0.56*
Among populations within species	259.12	21	1.84	38.15	Φ*_S__C_* = 0.87*
Within populations	41.98	159	0.26	5.49	Φ*_S__T_* = 0.95*
*C. chinensis* var. *brevisepala*					
Among populations	158.43	3	4.89	91.78	Φ*_ST_* = 0.92*
Within populations	18.85	43	0.44	8.23	
*C. chinensis* var. *chinensis*					
Among populations	22.51	4	0.93	75.33	Φ*_ST_* = 0.75*
Within populations	7.29	24	0.30	24.67	
*C. deltoidea*					
Among populations	27.26	4	1.12	88.43	Φ*_ST_* = 0.88*
Within populations	3.68	25	0.15	11.57	
*C. omeiensis*					
Among populations	37.38	6	0.97	81.20	Φ*_ST_* = 0.81*
Within populations	8.57	38	0.23	18.80	
*C. teeta*					
Among populations	13.54	4	0.58	78.74	Φ*_ST_* = 0.79*
Within populations	3.60	23	0.16	21.26	

*P < 0.05. The bold and red letter indicates that the sample is from cultivated.

### Phylogenetic Relationships of cpDNA Haplotypes and Divergence Time Estimates

**FIGURE 3:**
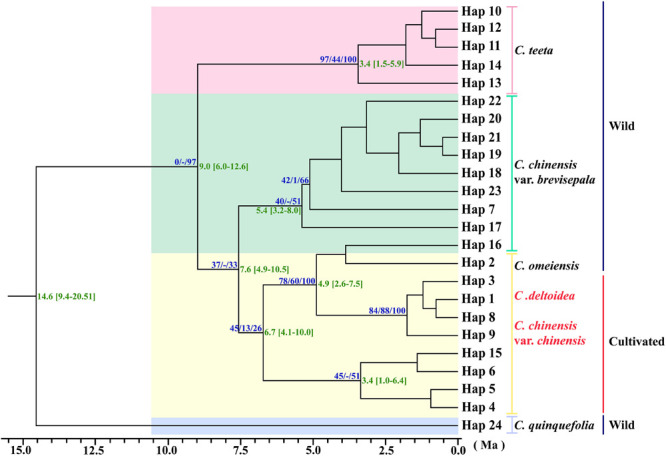
Phylogenetic relationships analysis using Maximum Parsimony (MP) methods based on cpDNA data. The numbers above the branches are bootstrap support values of Neighbor-Joining and Maximum parsimony tree, and Bayesian posterior probabilities (PPs) of 100% of BI tree (NJ/MP/BI) between major lineages. Time scale (with 95% HPD intervals in square brackets) are shown to the right of each node with green color. The red letter indicates that the sample is from cultivated.

**FIGURE 4:**
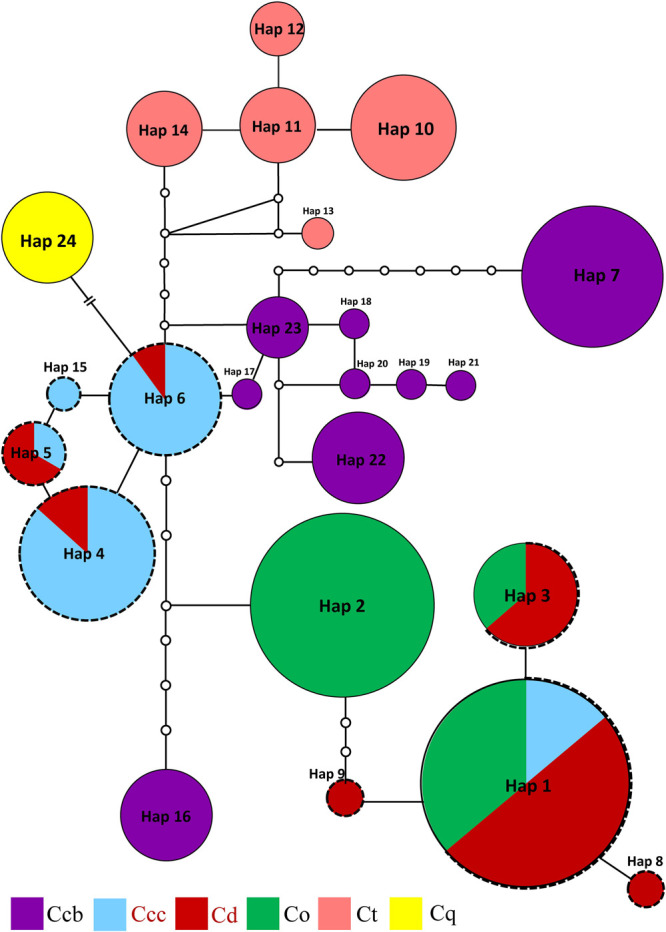
Parsimony network for all cpDNA haplotypes of *C. chinensis* var. *brevisepala* (Ccb), *C. chinensis* var. *chinensis* (Ccc), *C. deltoidea* (Cd), *C. omeiensis* (Co), *C. teeta* (Ct), and *C. quinquefolia* (Cq) species based on cpDNA data. The relative size of each circle corresponds to proportional to haplotype frequencies. The small hollow dots indicate hypothetical missing haplotypes. The network shown in different colors correspond to each species. The red letter and black dot line indicate that the sample is from cultivated.

Three phylogeny analysis methods (NJ, MP, BI) generated congruent tree topologies in terms of the major clades (Figure 3). Overall, the 24 cpDNA haplotypes were clustered into four groups corresponding to individual species. All haplotypes of cultivated Huang-lian and *C. omeiensis* (Hap1-6, 8-9, 15) were included in a monophyletic group with 37% (NJ) or 33% (BI) bootstrap support values, of which paraphyletic clade was the group contained all haplotypes of *C. chinensis* var. *brevisepala* except Hap16. Haplotypes of *C. teeta* (Hap 10-14) formed a monophyletic group with firm support. The haplotype of *C. quinquefolia* was distinctively apart. Similar results were depicted by the statistical parsimony network (Figure 4). Haplotypes of cultivated Huang-lian and *C. omeiensis* connected to form a network. Haplotypes of *C. chinensis* var. *brevisepala* formed a relatively independent clade network connected to Hap6 of cultivated Huang-lian except for Hap16 (Corresponding to the H10, H11, H140, H142, H148, H161 of ITS haplotypes). Haplotypes of *C. teeta* and *C. quinquefolia* grouped into two significant divergent clades with relatively far from the other four species by more than four mutational steps. This similar grouping patterns can also be obtained from statistical parsimony network results of ITS data (Figure 2). Haplotypes of *C. omeiensis* formed an intermediate network and shared H2 of ITS haplotypes with cultivated *C. deltoidea* and two haplotypes (H37 and H38 of ITS haplotypes) occupying tips belong to cultivated *C. deltoidea* (Figure 2).

Assuming substitution rates of 1.52 × 10^–9^ s/s/y, the divergence time of *C. chinensis* var. *brevisepala* populations and three species group (cultivated Huang-lian and *C. omeiensis*) was estimated as 7.6 Ma, with a 95% HPD confidence interval (95% CI) of 4.9–10.5 Ma (Figure 3). *C. teeta* diverged from the above four species earlier, about at 9.0 Ma (95% CI: 6.0−12.6 Ma). *C. quinquefolia* was firstly different from other species; the divergence time was estimated as 14.6 Ma (95% CI: 9.4−20.51 Ma). All results thus indicate that the divergence of *Coptis* species happened before Pleistocene (∼2.5–0.01 million years ago; Ma). Although no fossil record correction was used in this study, the results of the divergent time evaluation are similar to the previous study (Xiang et al., 2018).

### Demographic History and Mismatch Analyses

**FIGURE 5:**
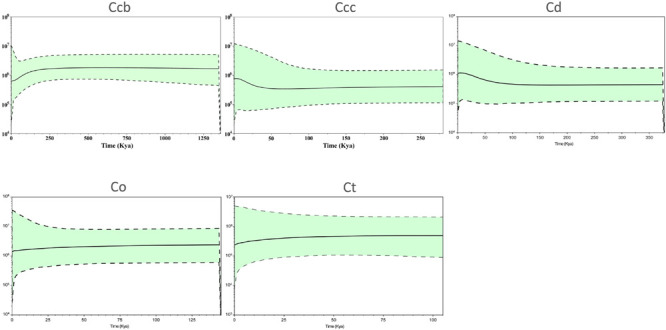
Bayesian skyline plot analysis for the effective population size over time for *C. chinensis* var. *brevisepala* (Ccb), *C. chinensis* var. *chinensis* (Ccc), *C. deltoidea* (Cd), *C. omeiensis* (Co), and *C. teeta* (Ct) species based on the cpDNA. The gray dash lines indicate 95 % confidence intervals.

**FIGURE 6:**
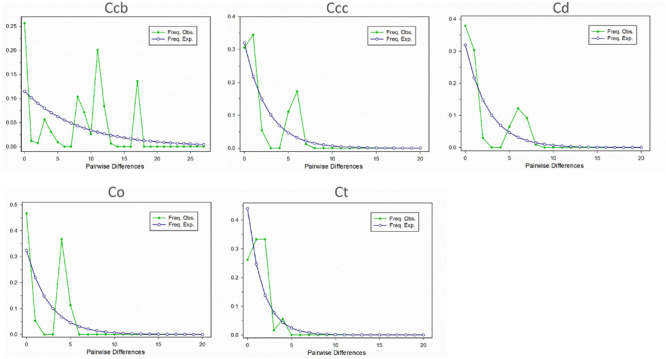
Mismatch distribution for cpDNA sequence data in *C. chinensis* var. *brevisepala* (Ccb), *C. chinensis* var. *chinensis* (Ccc), *C. deltoidea* (Cd), *C. omeiensis* (Co), and *C. teeta* (Ct) species, respectively. The line with hollow dot represents expected values and solid dot represents observed values.

A Bayesian skyline plot showed recent population size rise in the populations of cultivated Huang-lian (*C. chinensis* var. *chinensis* and *C. deltoidea*) and decline (*C. chinensis* var. *brevisepala*) or constant (*C. omeiensis* and *C. teeta*) in the populations of wild *Coptis* species (Figure 5). The cpDNA sequence mismatch analysis results for five species’ populations displayed a multimodal distribution pattern (Figure 6). There were nearly no significant SSD statistics and the *H*_Rag_-value under the demographic expansion and spatial expansion models (Table 6). All these observations indicated the absence of an expansion event. However, whether the values of Tajima’s *D* and Fu and Li’s *D*^∗^ was positive or negative differed among species and populations (Table 2), which implied that demographic expansion existed in certain groups.

**TABLE 6:** Summary of mismatch distribution analysis (parameters of demographic and spatial expansion) of chloroplast DNA sequence data based on all populations of *C. chinensis* var. *brevisepala*, *C. chinensis* var. *chinensis*, *C. deltoidea*, *C. omeiensis* and *C. teeta*, respectively.

Species	Population	Demographic expansion		Spatial expansion	
		SSD	*H*_Rag_	SSD	*H*_Rag_
*C. chinensis* var. *brevisepala*					
	Ccb(HJ)W	0.09*	0.69*	0.05*	0.69*
	Ccb(SX)W	–	–	–	–
	Ccb(JGS)W	0.10*	0.23*	0.02*	0.23*
	Ccb(TG)W	–	–	–	–
*C. chinensis* var. *chinensis*					
	Ccc(EMS1)C	0.11*	0.53*	0.11*	0.53*
	Ccc(EMS2)C	–	–	–	–
	Ccc(XE)C	0.01*	0.14*	0.01*	0.14*
	Ccc(JFS)C	0.04*	0.35*	0.04*	0.35*
	Ccc(DZ)C	0.05*	0.40*	0.05*	0.40*
*C. deltoidea*					
	Cd(EMS1)C	0.07*	0.36*	0.07*	0.36*
	Cd(EMS2)C	–	–	–	–
	Cd(HY)C	0.01*	0.20*	0.01*	0.20*
	Cd(EMS3)C	–	–	–	–
	Cd(EMS4)C	0.28*	0.31*	0.00*	0.31*
*C. omeiensis*					
	Co(EMS1)W	–	–	–	–
	Co(EMS2)W	–	–	–	–
	Co(EMS3)W	–	–	–	–
	Co(EMS4)W	–	–	–	–
	Co(EMS5)W	–	–	–	–
	Co(EMS6)W	–	–	–	–
	Co(EMS7)W	0.36*	0.84*	0.23*	0.84*
*C. teeta*					
	Ct(FG3)C	–	–	–	–
	Ct(GLGS1)C	–	–	–	–
	Ct(GLGS2)W	0.05	0.40	0.05	0.40
	Ct(FG1)W	–	–	–	–
	Ct(FG2)C	0.20	0.68	0.10	0.68
*C. quinquefolia*					
	Cq(YL)W	–	–	–	–

*SSD: sum of squared deviations; *H*_R__ag_: the Harpending’s Raggedness index. Asterisks indicate values with associated *P*-values < 0.05; -: The variance of the mismatch distribution analysis is too small; no demographic parameters can be estimated. The bold and red letter indicates that the sample is from cultivated.*

### Historical Biogeography Inference

**FIGURE 7:**
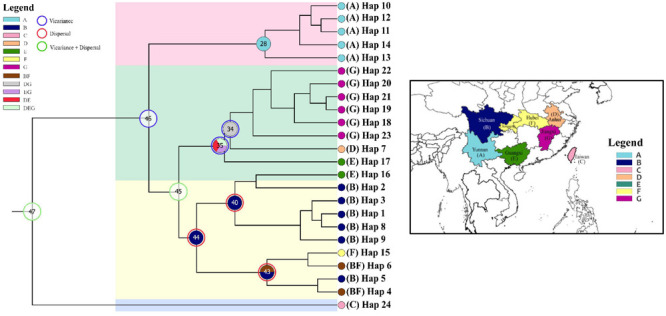
Ancestral distributions reconstructed and dispersal–vicariance analysis generated by RASP from the cpDNA data. Map showing seven geographical regions in colors as defined in RASP analysis. Pie charts on nodes display the relative probabilities of possible ancestral ranges.

Ancestral ranges were obtained to infer vicariance and dispersal events in *Coptis* species by RASP analysis using plastid DNA data (Figure 7 and Table 7). Results support three dispersal events (node 44, 43 and 40) for the groups of two cultivated Huang-lian (*C. chinensis* var. *chinensis* and *C. deltoidea*) and *C. omeiensis*. The ancestor of the three species first colonized Sichuan (B), which was followed by one dispersal event, from Sichuan (B) to Chongqing and Hubei (F). Furthermore, dispersal + vicariance event was detected between the ancestor of the three species and *C. chinensis* var. *brevisepala* species on node 45, which indicates gene flow among them. However, distinctive vicariance signals (node 35 and 34) were observed in *C. chinensis* var. *brevisepala*, of which ancestor originated on Anhui (D), Jiangxi (G) and Guangxi (E), respectively, and several vicariance events occurring among these ancestor regions. Meanwhile, a vicariance event was also evident between *C. teeta* and the above four species (node 46), suggesting the independent origin of *C. teeta*.

**TABLE 7:** The ancestral areas and dispersal-vicariance analysis inferred through RASP based on cpDNA data.

Node	Ancestral areas	RASP ROUTE	Dis	Vic	Prob
28	[A]	A->A^A->A| A	0	0	1.00
34	[DG]	DG->D| G	0	1	1.00
35	[DEG| EG| DE]	DEG->E| DG	0	1	0.33
40	[B]	B->B^B->BE^B->B| BE	1	0	1.00
43	[BF| B]	BF->BF^B->B| BF	1	0	0.25
44	[B]	B->B^B->BF^B->BF| B	1	0	0.50
45	[BDG| BEG| BDEG| BG| BD| BE| BDE]	BDG->BDEG->B| DEG	1	1	0.05
46	[ABDG| ADG| AEG| ADEG| ABEG| ABE| ABD| ADE| ABG| ABDE]	ABDG->BDG| A	0	1	0.14
47	[BCDG| ACDG| ACEG| CDEG| BCEG| ABCE| ABCD| ACDE| ABCG| BCDE]	BCDG->CABDG->C| ABDG	1	1	0.01

*Ancestral areas for the node and the number of dispersal (Dis) and vicariance (Vic) events is shown.*

## Discussion

The results of plant domestication were shaped by selection resulting from planting practices, human preferences, and agricultural environments, as well as the relevant population genetic processes following the reduction of the adequate population size (Smýkal et al., 2018). The domestication of wild plants always leads to genetic bottlenecks, and thus results in reduced genetic diversity due to founder effects and unintentional or intentional selections (Doebley et al., 2006). The analysis revealed that the genetic diversity of cultivated Huang-lian (*C. chinensis* var. *chinensis* and *C. deltoidea*) has not reduced compared with three wild *Coptis* species. The proportion of the population with unique haplotypes in cultivated Huang-lian was lower than that in wild *Coptis* species. However, the genetic diversity within populations of cultivated Huang-lian raised. The wild species and cultivated species have similar levels of genetic diversity. The loss of unique genetic composition in cultivated species are also to be found on medicinal plants of *Scutellaria baicalensis* (Yuan et al., 2010).

General agricultural large-scale planting practices significantly reduced the adequate population size of cultivated crops and allowed genetic drift (Smýkal et al., 2018). There is much evidence that domestication results in reduced genetic diversity in several cultivated crops and medicinal plants (Hollingsworth et al., 2005; Lam et al., 2010; Gao et al., 2017; Zhang et al., 2017). Although these bottlenecks affect the genetic diversity in the domestication process, the population sizes of the census will increase when the domesticated species are planted across extended areas (Smýkal et al., 2018). Furthermore, if the expanding population encounters a co-existing wild relative in a new area, the domesticated species and the wild species may have admixture or interbreeding, accompanied by genetic introgression events, and the domesticated population may have additional genetic components. This process might counteract the low genetic diversity caused by bottleneck effects and natural selection (Larson et al., 2014; Hazzouri et al., 2015; Mathew et al., 2015). Crops with significant hybridization or introgression, such as North African dates or citrus, and in outcrossing perennials crops, the cultivated populations may maintain high genetic diversity or increase relative to their wild relatives (Miller and Gross, 2011; Smýkal et al., 2018). A genetic bottleneck occurs during domestication, or during the founding events period, the composition of the gene pools may be further altered when the domesticated species moved away from the center of origin (Smýkal et al., 2018).

In China, *C. chinensis* var. *chinensis* is planted in large areas (0.32 million mus) (Wei and Zhang, 2006). *C. deltoidea* is only cultivated in Emeishan and adjacent regions, which cultivation areas have less than 5 thousand mus. Wild *Coptis* of *C. omeiensis* is mainly distributed on Emeishan in Sichuan province, overlapping with the distribution of *C. deltoidea* and *C. chinensis* var. *chinensis*. Therefore, a genetic material exchange may occur between Large-scale planting of cultivated Huang-lian and wild *Coptis* species, resulting in no significant decline in the genetic diversity of cultivated Huang-lian. In the cpDNA results of this study, cultivated Huang-lian and wild *C. omeiensis* shared multiple haplotypes, which indicates the existence of gene flows. The *C. omeiensis* has the lowest haplotype diversity. *C. omeiensis* has small population size and low natural fertility problems, which may cause low genetic diversity of plastid sequences (Huang, 2012).

Plant domestication affects not only the genetic variation of cultivated populations but also the structural patterns of genetic variation (Miller and Schaal, 2006). Assessing the genetic differentiation structure of cultivated Huang-lian and wild *Coptis* species, the genetic variation among intraspecies populations of cultivated Huang-lian showed lower population differentiation and a certain degree of homogenization. The wild *Coptis* species of *C. quinquefolia* and *C. teeta* have significant divergence from other species of *Coptis*. Wild *Coptis* species of *C. chinensis* var. *brevisepala* had the highest genetic variation among populations. These genetic distribution patterns indicate that the gene flow among populations of cultivated Huang-lian is higher relative to wild species of *Coptis*. Furthermore, *C. chinensis* var. *chinensis* is propagated by seeds and no stolon (Song et al., 2010) which might indicate widespread seed change among populations in different planting areas during extensive cultivation. Shi et al. (2008) used ISSR molecular markers to estimate genetic variation and gene flow among wild and cultivated populations of *C. chinensis* var. *chinensis*, showing a similar degree of genetic diversity and high gene flow between wild and cultivated populations (Shi et al., 2008). These results can support the present-day cultivated *C. chinensis* var. *chinensis* may have been introduced from a large number of wild ancestral seeds. Therefore, seeds from the primary gene pool include most of the genetic composition of the wild populations.

Moreover, the results of the demographic history analyses showed a recent population size rise in the populations of cultivated Huang-lian (Figure 5). This evidence also can infer that domesticated Huang-lian from abundant wild populations resources and have been domesticated and cultivated from multiple places through multiple events, resulting in a large initial population size of cultivated Huang-lian. Furthermore, historical biogeography inference also revealed multiple dispersal events in the groups of two cultivated Huang-lian (Figure 7 and Table 7). Additionally, mismatch distribution analysis showed a multimodal curve of pairwise differences which corresponds to no evident expansion and a recent bottleneck (Figure 6; Ray et al., 2003; Liao et al., 2010). However, neutrality tests were positive or negative differed among species and populations (Table 2). These analyses indicated that demographic expansion existed in specific groups and some groups experienced bottlenecks.

Compared with cultivated Huang-lian, wild relatives *Coptis* species have a high genetic differentiation among populations, indicating limited gene flow among populations. Wild *Coptis* species in China are facing habitat fragmentation crisis due to human over-exploitation and habitat demolition, causing some wild *Coptis* species are in an endangered crisis (Saha et al., 2015; Zhang et al., 2018). The Bayesian skyline plot also showed the recent population decline of certain wild *Coptis* species. However, the haplotype distribution results show that there are abundant unique haplotype resources in wild *Coptis* populations, which is very important for genetic improvement and conservation of domesticated species. Therefore, the intervention of conservation strategies is more important to maintain the genetic diversity of domesticated species.

The distribution of *C. omeiensis* overlaps with cultivated Huang-lian. At present, the cultivation practices of Huang-lian is mainly cultivated by traditional agriculture practices. Farmers plant Huang-lian in wild habitat, courtyard or semi-wild farming. Traditional agricultural practices systems can effectively maintain considerable genetic diversity resources has been proposed (Altieri, 2004; He et al., 2009; Guo et al., 2010; Yuan et al., 2010; Ren et al., 2018). These factors explain the close relationship between cultivated Huang-lian and *C. omeiensis*, and also indicate that *C. deltoidea* preserves considerable genetic resources of wild species. Moreover, it also explained the genetic diversity significant increase within the populations of cultivated Huang-lian. *C. omeiensis* was called as Emei wild Huang-lian and *C. deltoidea* was called as Emei domestic Huang-lian. Meanwhile, *C. omeiensis* was always used as an alternative Chinese herbal medicine for Huang-lian because their medicinal constituents are similar (Chen et al., 2017).

According to the ITS and cpDNA parsimony network information, *C. chinensis* var. *chinensis* is more closely related to *C. chinensis* var. *brevisepala* than *C. omeiensis* but does not form a monophyletic group. All haplotypes of cultivated Huang-lian and *C. omeiensis* were formed a monophyletic group (Figure 3), of which clustering the group contained all haplotypes of *C. chinensis* var. *brevisepala* except Hap16 of cpDNA haplotype. This similar pattern can also be obtained from the ITS parsimony network (Figure 2). Phylogenetic discernment between related taxa or between populations may be difficult because of the incomplete lineage sorting (Hollingsworth et al., 2011) or recently gene flow (Aguirre-Liguori et al., 2019). The sample contained in Hap16 corresponded to H10, H11, H140, H142, H148, H161 of ITS haplotypes (Figure 2 and Supplementary Table S2). The cultivated Huang-lian and *C. chinensis* var. *brevisepala* shared H30 of ITS haplotypes. From the ITS and cpDNA parsimony network showed that *C. chinensis* var. *brevisepala* has two genetic groups (Figures 2, 4). One genetic group is Hap16, corresponding to ITS H10, H11, H140, H142, H148, H161, and the second genetic group is the remaining Ccb haplotypes. The divergence time of *C. chinensis* var. *brevisepala* and *C. chinensis* var. *chinensis* (7.6 Ma) is earlier than the group of *C. chinensis* var. *chinensis*, *C. deltoidea* and *C. omeiensis* (Figure 3). According to biogeographic analysis, dispersal + vicariance event was detected between the ancestor of the cultivated Huang-lian and *C. chinensis* var. *brevisepala* species, which indicates gene flow among them and cause the two genetic group of *C. chinensis* var. *brevisepala*. However, several vicariance events occurring among *C. chinensis* var. *brevisepala*. *C. chinensis* var. *brevisepala* has abundant unique haplotypes, yet the restricted gene flow and vicariance among populations emphasize the importance of conserving genetic resources of wild relatives.

Understanding the natural genetic diversity and structure within and among populations of a species is essential for developing suitable conservation strategies to protect genetic resources and genetic improvements. Medicinal plants are a valuable source of herbal drug products. Proper assessment of medicinal plant genetic resources is useful information for developing conservation plans for protecting genetic diversity (Falk and Holsinger, 1991). Genetic information before domestication might have been retained in wild populations. According to haplotype distribution information, there are abundant unique genetic resources in wild relatives *Coptis* species. However, most wild relatives *Coptis* species of cultivated Huang-lian in China are facing an endangered crisis and are influenced by human activities. Thus, protection measures must be taken to avoid further reduction of wild *Coptis* species genetic resources. Currently, large-scale cultivation of cultivated Huang-lian becomes more wild populations go extinct. Restricted gene flow and population size decline in wild *Coptis* species highlight the importance of *in situ* and *ex-situ* conservation. *Ex-situ* conservation rerelease threatened species. *Ex-situ* focuses on the use of traditional and modern breeding techniques to maintain the genetic diversity of species. Regardless of the conservation strategies and resource management, the sustainable use of medicinal plant resources should be fully considered. The application of biotechnical approaches could help improve yield and modify the efficacy of medicinal plants (Kasagana and Karumuri, 2011; Chen et al., 2016). In this study, comprehensively evaluates the genetic resources of wild *Coptis* species and cultivated Huang-lian, insight into the genetic effects of long-term artificial selection on medicinal plants. This study provides practical perspectives to genetic resource conservation efforts and management for cultivated Huang-lian and relatives *Coptis* species.

## Data Availability Statement

The datasets generated for this study can be found in the all information list on Supplementary Table S1.

## Author Contributions

Q-JY, Y-CC, and L-QH conceived and designed the experiments. XW, X-QL, Y-ZK, X-LJ, J-HS, Z-YZ, Q-JY, and Y-CC performed the experiments. Y-ZK, X-LJ, Q-JY, and Y-CC analyzed the data. XW, X-QL, Y-ZK, X-LJ, J-HS, Z-YZ, Q-JY, and Y-CC contributed reagents, materials, and analysis tools. Y-ZK, Q-JY, Y-CC, and L-QH wrote the manuscript. XW, X-QL, Y-ZK, X-LJ, J-HS, Z-YZ, Q-JY, Y-CC, and L-QH conceived of the study, edited the manuscript, and approved the final manuscript. All authors contributed to the article and approved the submitted version.

## Conflict of Interest

The authors declare that the research was conducted in the absence of any commercial or financial relationships that could be construed as a potential conflict of interest.
