# Supplementary material for: Genetic Diversity and Phylogeography of the Important Medical Herb, Cultivated Huang-Lian Populations, and the Wild Relatives Coptis Species in China
Source: Front Genet. 2020 Jul 3;11:708. doi: 10.3389/fgene.2020.00708 (PMC7350934; doi:10.3389/fgene.2020.00708)
Supplement: Supplementary file 1 [file Table_1.DOCX]

**Table S1The information on the ploidy level, propagation, cultivation status of six *Coptis* species.**

| Species | Distribution | Ploidy level | Type of propagation | Cultivated/ wild statue | Official/folk medicinal materials |
| --- | --- | --- | --- | --- | --- |
| *C. chinensis var. chinensis* | Chongqing, Sichuan, Hubei, Hunan, Guizhou, Shaanxi | Diploid  (2n = 2x = 18) | Seeds | Wild, Cultivated | Official |
| *C. deltoidea* | Sichuan  (Emeishan, Hongya county) | Triploid  (2n = 3x = 27) | Stolons | Wild, Cultivated | Official |
| *C. chinensis var. brevisepala* | Guangxi, Guangdong, Anhui, Fujian, Zhejiang | Diploid  (2n = 2x = 18) | Seeds | Wild | Folk |
| *C. omeiensis* | Henan, Sichuan (Emeishan and adjacent regions) | diploid  (2n = 2x = 18) | Seeds | Wild | Folk |
| *C. teeta* | Yunnan, Xizang, Eastern Himalaya | diploid  (2n = 2x = 18) | Seeds and Stolons | Wild, Cultivated | Official |
| *C. quinquesecta* | Yunnan (Jinping county) | Diploid  (2n = 2x = 18) | Seeds | Wild | Folk |
| *C. quinquefolia* | Taiwan | Diploid  (2n = 2x = 18) | Seeds | Wild | Folk |
