# Supplementary material for: Genetic Diversity and Phylogeography of the Important Medical Herb, Cultivated Huang-Lian Populations, and the Wild Relatives Coptis Species in China
Source: Front Genet. 2020 Jul 3;11:708. doi: 10.3389/fgene.2020.00708 (PMC7350934; doi:10.3389/fgene.2020.00708)
Supplement: Supplementary file 2 [file Table_2.DOCX]

**Table S2 Details of haplotypes composition in each population and amount of each cpDNA (H1–H24) and ITS (H1–H158) haplotypes in 27 populations.**

| Taxon | Population code | cpDNA haplotypes  (no. of individuals) | ITS haplotypes  (no. of clones) | GenBank Accession no. | | | |
| --- | --- | --- | --- | --- | --- | --- | --- |
|  |  |  |  | *mat*K | *rbc*L | *trn*H-*psb*A | ITS |
| ***C. chinensis* var. *brevisepal****a* | Ccb(HJ)W | Hap16(7)、Hap17(1) | H10(8)、H11(1)、H30(20)、H140(6)、H141(1)、H142(1)、H143(1)、H144(2)、H145(1)、H146(1)、H147(3)、H148(1)、H158(3)、H159(1)、H160(1)、H161(1)、H162(2) | KY780755 | KY780756、KY780758 | KY780760 | KY780723-KY780725、  KY780729-KY780730、  KY780733-  KY780743 |
|  | Ccb(SX)W | Hap7(21) | H29(2)、H31(1)、H174(21)、H86(1) 、H99(1)、H100(1)、H101(1)、H102(2)、H103(1)、H104(1)、H105(1)、H106(1)、H107(1) | KY780752 | KY780758 | KY780759 | KY780726 |
|  | Ccb(JGS)W | Hap18(1)、Hap19(1)、Hap20(1)、Hap21(1)、Hap22(9) | H169(6)、H170(1) | KY780753 | KY780757、 KY780758 | KY780761-  KY780765 | KY780746、KY780747 |
|  | Ccb(TG)W | Hap23(5) | H171(2)、H172(2)、H173(2)、 | KY780754 | KY780758 | KY780766 | KY780748-KY780750 |
| ***C. chinensis* var. *chinensis*** | Ccc(EMS1)C | Hap4(2)、Hap5(1)、Hap6(1) | H1(9) | KY780713、KY780714 | KY780715 | KY780717、  KY780718、KY780721 | KY780655 |
|  | Ccc(EMS2)C | Hap1(5) | H14(4)、H19(1)、H21(10)、H22(1)、H23(5)、H24(1)、H25(1)、H26(1)、H50(1)、H58(2)、H59(1)、H60(1)、H60(1)、H62(1)、H63(1)、H64(1)、H65(2)、H66(1)、H79(1)、H128(1)、H129(1)、H130(1)、H131 (1)、H152(1)、H153(3)、H154(1)、H155(1) | KY780712 | KY780715 | KY780719 | KY780656-  KY780657  KY780660-  KY780666  KY780691-KY780708 |
|  | Ccc(XE)C | Hap4(4)、Hap6(2)、Hap15(1) | H1(2)、H29(1)、H30(4)、H80(1)、H81(1)、H(1)、H83(1)、H84(1)、H85(1)、H86(3)、H87(1)、H88(1)、H89(4)、H90(1)、H91(1)、H92(1)、H93(1)、H94(1)、H95(1)、H96(1)、H97(1)、H98(1) | KY780713、KY780714 | KY780716 | KY780717、KY780718、KY780721 | KY780668-  KY780684  KY780686-  KY780689 |
|  | Ccc(JFS)C | Hap4(4)、Hap6(3) | H1(8) | KY780713、KY780714 | KY780715 | KY780720、  KY780721、KY780722 | KY780685 |
|  | Ccc(DZ)C | Hap4(3)、Hap6(3) | **-** | KY780713、KY780714 | KY780715 | KY780717、  KY780718、KY780722 |  |
| ***C. deltoidea*** | Cd(EMS1)C | Hap4(2)、Hap5(2)、Hap6(1) | H1(9) | KY780863、KY780864 | KY780867 | KY780868、  KY780869、KY780872 | KY780767 |
|  | Cd(EMS2)C | Hap1(7) | H14(2)、H15(1)、H16(1)、H17(1)、H18(2)、H19(2)、H20(2)、H21(8)、H22(1)、H23(5)、H27(1)、H28(1)、H39(1)、H40(1)、H41(1)、H42(1)、H43(1)、H44(1)、H45(1)、H46(1)、H47(3)、H58(2)、H65(2)、H66(1)、H108(1)、H109(1)、H110(1)、H111(1)、H112(1)、H113(1)、H114(1)、H115(1)、H116(1)、H117(1)、H118(1)、H119(1)、H149(1)、H150(1)、H151(1) | KY780862 | KY780866、 KY780867 | KY780871 | KY780774-KY780780、  KY780788-KY780796、  KY780822-KY780835、  KY780851-KY780859 |
|  | Cd(HY)C | Hap1(4)、Hap8(1) | H3(3)、H19(3)、H20(1)、H21(8)、H22(1)、H23(1)、H47(2)、H48(1)、H49(1)、H50(2)、H51(1)、H52(1)、H53(1)、H54(1)、H55(1)、H56(1)、H57(1)、H65(1)、H120(1)、H121(1)、H122(1)、H123(1)、H124(1)、H125(1)、H126(1)、H127(1) | KY780861、KY780862 | KY780867 | KY780870、KY780871 | KY780797-KY780800、  KY780802-  KY780807、  KY780836-KY780849、  KY780855-KY780856、  KY780773 |
|  | Cd(EMS3)C | Hap3(5) | H2(11)、H32(1)、H33(1)、H34(1)、H35(1)、H36(1)、H37(1)、H38(1) | KY780860 | KY780867 | KY780871 | KY780769-KY780771、KY780782-KY780786 |
|  | Cd(EMS4)C | Hap1(7)、Hap9(1) | H3(1)、H21(1)、H23(1)、H47(2)、H55(1)、H65(1)、H66(1)、H67(1)、H68(1)、H69(1)、H70(1)、H71(1)、H72(1)、H73(1)、H74(1)、H75(1)、H76(1)、H77(1)、H78(1)、H79(1) | KY780862 | KY780865 | KY780871 | KY780808-KY780821、  KY780849-KY780852、  KY780855 |
| ***C. omeiensis*** | Co(EMS1)W | Hap1(5) | H2(6) | KY780890 | KY780893 | KY780895 | KY780886 |
|  | Co(EMS2)W | Hap2(13) | H2(16)、H12(1)、H13(1)、H132(1)、H133(7)、H134(1)、H135(1)、H136(1)、H137(1)、H138(1)、H139(1)、H156(1)、H157(1) | KY780891 | KY780892 | KY780894 | KY780873-  KY780874、  KY780876-  KY780886 |
|  | Co(EMS3)W | Hap2(6) | H2(7) | KY780891 | KY780892 | KY780894 | KY780886 |
|  | Co(EMS4)W | Hap1(8) | H2(11) | KY780890 | KY780893 | KY780895 | KY780887 |
|  | Co(EMS5)W | Hap2(3) | H2(13) | KY780891 | KY780892 | KY780894 | KY780886 |
|  | Co(EMS6)W | Hap2(3) | H2(5) | KY780891 | KY780892 | KY780894 | KY780887 |
|  | Co(EMS7)W | Hap2(3)、Hap3(4) | H2(10) | KY780889、KY780891 | KY780892 | KY780894、  KY780895 | KY780886 |
| ***C. teeta*** | Ct(FG3)C | Hap10(5) | H4(6)、H5(4) | KY780919 | KY780921 | KY780922 | KY780902、 KY780909 |
|  | Ct(GLGS1)C | Hap10(7) | H5(5) | KY780919 | KY780921 | KY780922 | KY780901 |
|  | Ct(GLGS2)W | Hap11(2)、Hap12(3) | H4(5) | KY780918 | KY780921 | KY780923、KY780927 | KY780904 |
|  | Ct(FG1)W | Hap14(6) | H4(4)、H6(15)、H9(1)、H163(9)、H164(2)、H165(1)、H166(1)、H167(1) | KY780918 | KY780921 | KY780927 | KY780905-KY780906、  KY780910-KY780915 |
|  | Ct(FG2)C | Hap11(4)、Hap13(1) | H6(2)、H7(1)、H8(1)、H9(1) | KY780919 | KY780920 | KY780924、  KY780925、KY780926 | KY780907、KY780908、  KY780911、KY780917 |
| ***C. quinquefolia*** | Cq(YL)W | Hap24(7) | H168(10) | KY780898 | KY780899 | KY780900 | KY780896 |
