# Supplementary material for: Genetic Diversity and Phylogeography of the Important Medical Herb, Cultivated Huang-Lian Populations, and the Wild Relatives Coptis Species in China
Source: Front Genet. 2020 Jul 3;11:708. doi: 10.3389/fgene.2020.00708 (PMC7350934; doi:10.3389/fgene.2020.00708)
Supplement: Supplementary file 3 [file Table_3.DOCX]

**Table S3 The number of ITS haplotypes shared among six *Coptis* species.**

|  | Ccc | Cd | Co | Ccb | Ct | Cq |
| --- | --- | --- | --- | --- | --- | --- |
| H1 | 19 | 9 | - | - | - | - |
| H2 | - | 11 | 68 | - | - | - |
| H14 | 4 | 2 | - | - | - | - |
| H19 | 1 | 5 | - | - | - | - |
| H21 | 5 | 17 | - | - | - | - |
| H22 | 1 | 2 | - | - | - | - |
| H23 | 5 | 7 | - | - | - | - |
| H30 | 4 | - | - | 20 | - | - |
| H50 | 1 | 4 | - |  | - | - |
| H58 | 2 | 3 | - | - | - | - |
| H65 | 2 | 4 | - | - | - | - |
| H66 | 1 | 1 | - | - | - | - |
| H79 | 1 | 1 | - | - | - | - |
